# Bouncing Beyond Adversity in Oncology: An Exploratory Study of the Association Between Professional Team Resilience at Work and Work-Related Sense of Coherence

**DOI:** 10.3390/curroncol31110537

**Published:** 2024-11-17

**Authors:** Dominique Tremblay, Djamal Berbiche, Mathieu Roy, Catherine Prady, Marie-José Durand, Marjolaine Landry, Sylvie Lessard

**Affiliations:** 1 Faculty of Medicine and Health Sciences, Université de Sherbrooke, Longueuil, QC J4K 0A8, Canada; djamal.berbiche@usherbrooke.ca (D.B.); catherine.prady.med@ssss.gouv.qc.ca (C.P.); marie-jose.durand@usherbrooke.ca (M.-J.D.); 2 Centre de Recherche Charles-Le Moyne, Longueuil, QC J4K 0A8, Canada; sylvie.lessard@usherbrooke.ca; 3 Institut National de Santé Publique du Québec, Montréal, QC H2P 1E2, Canada; mathieu.roy7@usherbrooke.ca; 4 Faculty of Medicine and Health Sciences, Université de Sherbrooke, Sherbrooke, QC J1H 5N4, Canada; 5 Department of Nursing, Université du Québec à Trois-Rivières, Drummondville, QC J2C 0R5, Canada; marjolaine.landry@uqtr.ca

**Keywords:** team resilience, adversity, sense of coherence at work, oncology, interdisciplinary team, quantitative research

## Abstract

Team resilience at work (TR@W) is an important resource for bouncing beyond adverse situations. Adopting a health-promoting salutogenic approach, this cross-sectional study explores whether oncology team resilience, which is significantly associated with work-related sense of coherence (Work-SoC), and examines the roles of team member characteristics, quality of work life, and perceived impact of COVID-19. Team members (*n* = 189) from four oncology settings in Québec (Canada) completed self-administered e-questionnaires. Structural equation modeling was used to identify the best-fitting model and significant relationships among study variables. The results showed a significant positive reciprocal relationship between TR@W and Work-SoC (R = 0.20) and between Work-SoC and TR@W (R = 0.39). These two variables were influenced by gender, gender roles, age, or COVID-19. The resulting model confirms our initial assumption that a higher level of TR@W is significantly associated with a more positive Work-SoC. Our findings provide new insights into subscale items perceived positively by oncology team members, such as perseverance, connectedness, and capability; and identify areas, such as self-care, within the team that may require greater attention to bounce beyond adversity. They also suggest there may be different levels (individual, team, and organizational) of resources under the health salutogenic umbrella.

## 1. Introduction

Team resilience at work refers to “the capacity of a group of employees to collectively manage the everyday pressure of work and remain healthy; to adapt to change and to be proactive in positioning for future challenges” [1] (p. 14). Given the seriousness, complexity, and rapid evolution of cancer care, nurses provide and coordinate patient-centered care within a wide variety of interdisciplinary team models, which may include oncologists, pharmacists, psychologists, social workers, and others [2]. Oncology teams carry out their work in an unprecedented challenging environment [3]. During COVID-19 rebound years, clinicians, managers, and support staff continue to experience additional challenges as growing caseloads and time pressures combine with workforce shortages, increasing administrative burdens and breakdowns in communication and coordination across the cancer trajectory [4,5,6]. The unavoidable impacts of cancer on the whole life of people living with and beyond cancer has shifted professional practice from individuals to interdependent teamwork and partnership with patients and families [7]. While interdependency is key for effective oncology teamwork [8], the COVID-19 pandemic fundamentally shifted professional work toward the obligation to manage one’s own practice, leading to struggles with the established dynamics of team functioning [9,10,11]. At the organizational level, network-based structures [12] and collaborative governance [7,13] remain underdeveloped. This constrains the capacity to face the volatility, uncertainty, complexity, and ambiguity of cancer care that can compromise patient outcomes and generate additional workload and burden for professionals [3,14,15].

Oncology teams use determination and creativity to contend with complex problems in a “system in crisis” [16] and offer person-centered care, but the effort can put their physical and mental health at risk [17]. From a system perspective, oncology teams confront national cancer programs focused on performance and are expected to achieve top-down objectives that may be at odds with grassroots team functioning [16,18]. Oncology teams are, to varying degrees, living these entangled adverse situations that contribute to an increased incidence of burnout and mental health problems in cancer care professionals [19], particularly in women oncologists and nurses [20]. The pandemic has only worsened these adverse situations. The accumulation of both chronic and acute adversity qualifies as a so-called wicked problem characterized by blurred definition, where there are multiple people with vested and mostly competing values, and where the evolving dynamics in the system are confusing [21]. Scholars highlight that experience of adversity is essential for teams to build resilience [22]. These “wicked problems” [21] raise a number of questions, making oncology teams a fertile ground for understanding resilience at work and contributing new knowledge that has implications for care provided by nurses and other team members: Why do oncology teams not let adversity define them? How do oncology team members continue to find sense in their work? What is so important to them in this work?

These questions highlight the importance of understanding team resilience at work in oncology as more than a buzzword heard everywhere since the pandemic. Despite the lack of consistency in the definition of team resilience [23], this study chooses a pragmatic approach. Team resilience at work is defined as a non-linear and ongoing process of minimizing the impact of adversity, managing to bounce beyond and mending while learning for the future [24,25]. Bouncing beyond refers to the ability of teams to overcome difficult situations and return to previous functioning or emerge stronger as the result of a dynamic process [26]. Team resilience at work can be developed by emphasizing team resources and strengths in a context characterized by complexity and uncertainty [27], while avoiding the stigmatization of team members who are coping with distress, feelings of burnout, or maladaptive coping strategies [28]. Striving for effective and responsive care, team resilience at work in oncology can be viewed as a very important resource that helps to respond to a “noble calling” to care for those affected by cancer [17] and maintain a sense of coherence at work despite adversity [6]. We conceptualize this asset as a “generalized resistance resource” (GRR) within Antonovsky’s salutogenic model [29].

To the best of our knowledge, this is the first time that team resilience in the context of oncology care has been analyzed as a GRR in terms of its relation to the sense of coherence components. GRRs refer to biological, material, or psychosocial factors, bringing capacities to manage the stressors with which a person or a group have to cope [30]. The salutogenic model of health presumes a reciprocal and dynamic relationship between GRRs and sense of coherence. In the work-specific domain, sense of coherence is defined as the perceived comprehensibility, manageability, and meaningfulness of a person’s current work situation [31]. These three components of the so-called Work-SoC, applied to teamwork in oncology, refer to the cognitive aspect of perceived professional practice as structured, consistent, and clear; the extent to which team members perceive a fair balance between job demands and the resources they can access to face acute and chronic adverse situations or implement complex interventions [32]; and the emotional aspect of sharing a work situation considered worthy of both engagement and involvement. Work-SoC is influenced—and can be modified—by interactions between individuals or groups and by characteristics of the work context (e.g., structures, rules and protocols, and processes) [33]. Building on a salutogenic model [34], team resilience at work may provide team members with sets of perceived work experiences characterized by cohesion, participation in shaping outcomes, and a workload balance. However, there is a dearth of research into the possible reciprocity between team resilience at work and a work-related sense of coherence in oncology teams.

This study explores whether team resilience at work in oncology is associated with a work-related sense of coherence. We assume that a higher level of team resilience at work is associated with a more positive perception of the work-related sense of coherence as a means of not letting adversity define oncology teams. It also examines whether both team members’ and context characteristics influence the association between these two main variables.

## 2. Materials and Methods

### 2.1. Study Design

This exploratory study was part of a larger research project that aimed to better understand how a multi-component intervention improves resilience at work in oncology teams in Québec (Canada) [35]. The present study used a cross-sectional design [36] to analyze data on team resilience at work and work-related sense of coherence across a sample of oncology team members.

### 2.2. Participants

Participants were from four Integrated Health and Social Service Centres (IHSSC or IUHSSC when it includes a university center) embedded in the national cancer network. The National Cancer Program in Québec (Canada) is part of the Ministry of Health and Social Services, the governing authority of the publicly funded healthcare system. One of the key elements of the cancer program is interdisciplinary team-based care operationalized through relational and cognitive proximity within and between teams as part of a “network-of-networks” [12]. Oncology departments include interdisciplinary teams providing direct care (oncologists, nurses, pharmacists, social workers, psychologists, physical therapists, and nutritionists) supported by managers and clerical staff.

Participants were eligible to participate in the study if they were a health professional, a manager, or a support staff member in the oncology department; worked at least 20 h per week; and had worked in oncology without a long-term leave during the 12 months before data collection. A total of 209 team members returned the online questionnaire, of which 189 fully completed it and were included in the analysis. Using SEM Power Calculation by MacCallum et al. [37,38] and with a Goodness of Fit Index (GFI) of 0.88 as alternative hypothesis vs. 0.80 as null hypothesis and degrees of freedom of 71, a risk α of 0.05, we obtain a power of 1 − β = 0.99.

### 2.3. Procedures

Following ethics approval, the PI presented the study at regular oncology team meetings as a pre-notification before the distribution of the e-questionnaire [39]. A designated local collaborator held the list of eligible team members, e-mailed invitations to complete the questionnaire, and the research professional checked the eligibility of team members who manifested an interest in participating. If eligible, a unique login link was sent to access the e-consent and e-questionnaire. Two reminders at 7 and 14 days were transmitted after the initial e-mail to boost the response rate. The questionnaire did not include any questions asking for identifying details. All responses were anonymous and collected using an individual SurveyMonkey account. Data collection was performed from 21 February 2022 to 19 June 2023.

### 2.4. Measures

Team resilience at work was operationalized with the R@W Team Scale (TR@W) French version [40]. This questionnaire has 42 items measured on a 7-point scale (from 1 = strongly disagree to 7 = strongly agree, with reverse scoring for negatively phrased items). Items are grouped into seven subscales, all with an acceptable value of alpha; however, the value of the full scale over 0.90 may suggest that some items are redundant [41]. McEwen and Boyd developed and validated the TR@W scale among employees (*n* = 344) across government, private, and non-profit sectors [40]. The results for the full scale (42 items) show a mean score of 4.29 (SD = 0.83), while, together, the seven subscales explain 63% of the variance. To the best of our knowledge, only one study has previously used the TR@W in the healthcare sector, among registered nurses in long-term care homes in the province of Ontario, Canada (*n* = 306; mean score = 4.5; SD = 1.21) [42]. Minor adaptations to items were made to the French version to render questions more specific to the oncology setting, where it has not been used previously.

The Work-Related Sense of Coherence (Work-SoC-9) French version has 9 items grouped into three subscales: comprehensibility (4 items describing perceived work situation as structured, consistent, and clear), manageability (2 items describing perceived availability of resources to face work demands), and meaningfulness (3 items describing perception that work is worthy of involvement) [31]. The full scale has a Cronbach’s alpha of 0.83. Participants respond to items according to their current and general work situation on a 7-point differential semantic scale, ranging from positive to negative perception (e.g., meaningless–meaningful). Bauer and Jenny (2007) [43] suggest that working conditions directly affect Work-SoC. A systematic review of the literature reports that the general Sense of Coherence (SoC) scale has been used in studies among individual nurses in the work context of oncology [44]. To the best of our knowledge, only one study has used the Work-SoC-9 in oncology [45]. That study, undertaken in Switzerland, examines the association between the Work-SoC-9 and oncology nurses’ confidence to implement an intervention supporting self-management in cancer patients. The findings support those in the validity study of the Work-SoC-9 [33,46].

Sociodemographic and professional characteristics included age, gender, gender-related roles at work, education level, type of profession, work experience, work experience in the oncology team, work status, and role. Gender-related role at work was determined using the Labor Force Gender Index (LFGI), a four-item questionnaire deemed representative of the Canadian labor market dealing with social role rather than biological sex [47]. The construction of the LFGI represented the sum of scores for the components, resulting in a score ranging between 0 and 10 for each respondent. The quality of life at work was assessed with the QoLW Thermometer (scale 0–100): (0–25, red = problem zone; 26–50, yellow = needs-improvement zone; and 51–100, green = good-QoLW zone) [48]. The perceived impact of work adversity specific to the COVID-19 pandemic was measured on a visual analog scale (0 = no impact; 100 = most significant impact).

Table 1 presents the full scales and subscales, along with the related number of items and Cronbach’s alpha.

### 2.5. Statistical Analysis

Survey data were exported from SurveyMonkey to an Excel spreadsheet. Statistical analyses were conducted using SAS software, version 9.4 [49]. Results with a *p* < 0.05 were considered significant. Descriptive statistics per item and subscale we used to summarize the variables. Analyses stratified by gender (LFGI) were explored (given the high proportion of female nurses) to determine if gender-specific aspects influence the association between TR@W and Work-SoC. Internal consistency was determined for variables using standardized Cronbach’s alpha. Descriptive statistics (mean and standard deviation) served to examine distribution. Our sample size was large enough that we could use listwise deletion to handle missing data.

We used structural equation modeling (SEM), which is a combination of multiple regression and factor analysis that deals with measured and latent variables, to test complex models. We formulated a hypothetical model of relationships between TR@W and Work-SoC and sociodemographic factors as covariates and performed SEM to find relationships between variables. We used the standardized ß values to identify significant relationships between the variables. Some of these relationships were bidirectional. To evaluate model fit, we used the SRMR (Standardized Root Mean Squared Residual) as an absolute indicator of goodness of fit, as well as the RMSEA, CFI, GFI, and TLI as relative fit indicators with the conventional cut-off values (i.e., SRMR < 0.08; RMSEA < 0.08; CFI > 0.90; GFI > 0.90; TLI > 0.90).

## 3. Results

### 3.1. Response Rate

The response rate was 26%. A total of 209 respondents returned the questionnaires, and 20 were not included in the study because of missing data rates of more than 20%, leaving 189 questionnaires included in the study.

### 3.2. Participant Characteristics

Table 2 reports participant demographics, work history, and role description in the oncology team. The mean age was 42.63 years (SD = 10.28), and the most frequent survey categorizations were female (80.42%), nurses (39.26%), university education-level completed (61.41%), and less than 10 years of experience in oncology (59.52%). The Labor Force Gender Index was 4.67 (SD = 1.72).

### 3.3. Perceived Team Resilience at Work and Work-Related Sense of Coherence in Context

The present study’s Cronbach’s alpha values reported in Table 1 were 0.96 for the TR@W overall scale and 0.66 for the Work-SoC-9 overall scale, showing, respectively, very reliable or reliable levels [50]. Table 3 presents descriptive statistics and correlations between study variables. Participants reported high levels of team resilience at work (M = 4.87; SD = 1.09), with the highest score for capability (M = 5.42; SD = 1.18) and lowest score for self-care (M = 4.35; SD = 1.44). The work-related sense of coherence was also high (M = 5.42; SD = 1.25) with subscale meaningfulness (M = 5.98; SD = 1.14), comprehensibility (M = 4.88; SD = 1.09), and manageability (M = 4.64; SD = 1.06). Age was significantly and negatively related to TR@W full scale, but non-significantly positively related to Work-SoC-9. Quality of life at work was (M = 65.14; SD = 22.82), representing a positive perception, and it was significantly associated with the two main variables and all subscales. COVID-19 was significantly and negatively related to Work-SoC9 overall score, more specifically with comprehensibility, but no association was found with TR@W. LFGI was significantly and positively related to both variables.

### 3.4. Model Fit

**Figure 1:**
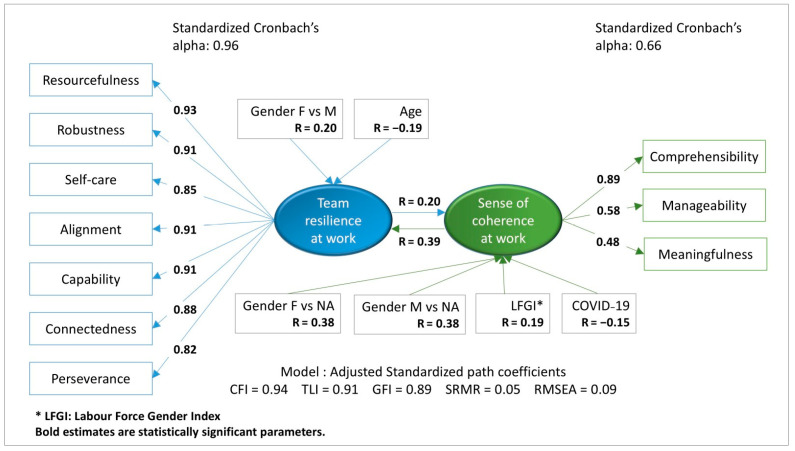
Model for team resilience at work (TR@W) and work-related sense of coherence (Work-SoC).

The model presented in Figure 1 shows that all TR@W subscales had a very high impact (standardized Cronbach’s alpha = 0.96), especially the resourcefulness subscale. The subscale best at explaining the Work-SoC was comprehensibility (R = 0.89). Strong team resilience at work allowed individuals to maintain a work-related sense of coherence. Standardized path coefficient values CFI = 0.94, TLI = 0.91, GFI = 0.89, SRMR = 0.05, and RMSEA = 0.09 showed a good adjustment of the observed relationships to the theoretical model. The first hypothesis was confirmed, showing a significantly positive relationship between the two main variables, TR@W and Work-SoC.

The analysis revealed a positive correlation between TR@W and gender female (R = 0.20) and between Work-SoC and Labor Force Gender Index (LFGI; R = 0.19), but it revealed a negative correlation between TR@W and age (R = −0.19) and between Work-SoC and perceived impact of COVID-19 on teamwork (R = −0.15). Moreover, our results showed a significant positive reciprocal relationship between TR@W and Work-SoC (R = 0.20) and between Work-SoC and TR@W (R = 0.39).

## 4. Discussion

### 4.1. Reciprocal and Positive Relationship Between TR@W and Work-SoC

This study explored the relationship between team resilience at work and work-related sense of coherence in the specific context of oncology. The findings confirmed our initial assumption of a positive relationship between these two variables. Our model appeared “good”, considering the CFI and TLI values of more than 0.90 and the SRMR at 0.05 [51]. This suggests that Antonovsky’s salutogenic model [29] is valuable in empirical understanding of the associations between oncology team resilience and the sense of coherence at work. Without denying the importance of pathogenic mental health risks, moral distress, and burnout seen in oncology [4], the salutogenic approach illuminated factors that could actively promote teams’ capacity to bounce beyond adversity situations. Additionally, the substantial correlations between these two variables and subscales described in Table 3 converged with the model of team resilience at work [52], which suggested that it has a function of sensemaking while team members problematize the situation, make sense of it together, and maintain or restore their teaming mechanisms (e.g., coordination, collaboration, and communication) to provide cancer care. Our unprecedented empirical demonstration in the specific context of oncology confirmed that team resilience has kinship with sense of coherence, quality of life, and interdisciplinarity under the salutogenic umbrella [53]. This would suggest that there may be different levels of GRR under the health salutogenic umbrella (e.g., individual, group, and organizational).

Going back to our question, why do oncology teams not let adversity define them, the three highest mean scores of the TR@W subscales suggested that perseverance was achieved through connectedness and that it improved capability. This revealed what Chatwal et al. (2023) [17] call the “noble calling” that stimulates job engagement and offers gratitude and meaning [33], and it was reflected in especially high meaningfulness scores in our study. The connectedness reflected that team members had to work together interdependently [54] and provide backup behaviors that characterize higher levels of interdisciplinary teamwork [54] bringing various perspectives and collective efforts to generate solutions to adversity-induced situations. These teaming processes were protected during the COVID-19 pandemic with the creation of “sanctuary” zones in oncology that were designed to protect patients with immunodeficiency and consequently maintained the team together, although comprehensibility was not always present.

The three other subscales revealed that the robustness needed to recover from the unexpected or to avoid quality-of-care failure despite adversity depended on team alignment and resourcefulness. These TR@W subscale scores that remained above 4.5 out of 7 may indicate that team members had confidence in their resources to bounce beyond and maintain focus on their role of being responsive to whole-person needs, even though they would have appreciated other resources outside the team. This required organizational agility and robust governance to overcome pessimism and find new ways of doing things when usual practices appeared neither feasible nor attractive. For example, telehomecare was introduced during the COVID-19 lockdowns without adequate planning but had since become a routine practice. Not surprisingly, the lowest scores were on the self-care subscale, supporting previous studies [40,42]. One strategy to develop a culture of self-care may be to develop awareness of coherent work experiences and reduce tensions between job resources and job demands [33]. At the collective level of the team, “team-care” involved a combination of socially supportive communication and backup behaviors from colleagues through sharing, supporting, and leading with compassion [55]. Although the fourth element of the Quadruple Aim in healthcare involves improving the work life and well-being of care teams [56], a real culture of promoting and deploying stress management routines and healthy work environments has yet to come. Attieh and Loiselle found that resilience was key to sustainable team functioning during COVID-19 [57]. However, they pointed to the scarcity of empirical studies on team resilience in oncology. Despite its limitations, the present study furthered efforts to raise awareness and better pinpoint to what extent team resilience and work-related sense of coherence are associated in this specialized healthcare domain.

### 4.2. Practical Implications

Our study results have several practical implications for maintaining or improving resilience as a GRR to cope with adversity situations in oncology teams. First, the multifaceted and dynamic nature of situations mean that there is no room for blame and that problems will never be “solved” once and for all. Faced with such “wicked problems” [21], developing a shared definition of problems is a starting point to finding innovative ways of aligning a myriad of nursing and healthcare professional roles to suit the local challenges. Drawing on each member’s talents, knowledge, skills, and responsibilities helps make available the team’s resources to provide people-centered responsive care [58]. Carefully balancing professional bounded autonomy and interdependency among nurses and other healthcare professionals helps avoid turf disputes along the cancer trajectory and reduce sources of stress. Positive teaming processes could prevent a loss of meaningfulness, voluntary (or not) renunciation, and “desilience”—the opposite of resilience [59].

Second, visible and committed managers who foster mutual trust and the expression of a plurality of points of view facilitate achievement of common goals that depend on team alignment and robustness, dimensions of resilience at work [40]. Deliberate support is needed for team connectedness and sense of belonging. The capacity for joint action reflects comprehensibility on multiple levels and dimensions and produces creative problem-solving dynamics, which in turn are associated with manageability and perseverance in achieving oncology team goals.

Third, in a deliberative multi-stakeholder symposium, attendees identified practical interventions aimed at enhancing resilience of professional care providers in oncology [6]. One of these was enhancing the articulation of evidence-based professional practice and patients’ experiential knowledge. This was described as a strategy to mitigate gaps in responsive cancer care by recognizing the patient’s role as a legitimate team partner. A second was raising awareness among policy leaders and decision makers of the importance of team resilience in oncology. Symposium attendees also recommended caution regarding the “tyranny of happiness” that resilience can impose. Placing responsibility for managing irreducible problems on the shoulders of individual healthcare professionals creates the risk of generating stigma around stress and burnout.

Fourth, a number of strategies have been shown to benefit dimensions that received the lowest scores in our study (self-care, alignment, and resourcefulness). These include mindfulness-based stress reduction, continuing education and training [60], reducing administrative burden and overtime [58], monitoring team member well-being and burnout metrics and providing resources [4], facilitating work-family balance, and allowing flexible schedules [4]. Although there is a need for more research evidence in oncology, innovative approaches may help team members. For example, the drama triangle framework suggests that people create their own stories to make sense of interpersonal relationships and their environment [61] and can become trapped in the cycle of victim (poor me), persecutor (blame others), and rescuer (elevated need to help). Understanding the role team members play may help people move beyond these stories and foster a constructive sense of coherence at work [62]. Another interesting avenue to maintain meaningfulness is the 3P’s framework known for its three areas of learned optimism: permanence (look at adversity as temporary or permanent), pervasiveness (how adversity affects your own and others’ lives), and personalization (adversity is your own fault or it just happens) [63].

Finally, the WHO undertook a thorough review of the role of the arts in improving health and well-being which opens new doors to resilience in healthcare settings [64]. Participatory arts classes, writing stories or keeping a diary, and drawing classes or art-appreciation classes have been found to enhance feelings of support in daily emotional challenges, help identify team issues for doctors and nurses, improve interdisciplinary teamwork, and increase tolerance for ambiguity. This review also reports that art activities can reduce exhaustion and death anxiety and increase emotional awareness in those working in end-of-life care. While interventions to optimize team resilience in oncology require more research work, there are feasible means of managing adversity and minimizing its impact. Translating these promising activities calls for mobilization at the individual, organizational, and policy level and to prioritize nurses’ and other team members’ health and well-being in an evolving system and society.

### 4.3. Limitations

One of the limits of the present study was the conceptual suitability of questionnaires that were not specifically designed for healthcare workplaces. We chose the TR@W because it addressed the capacity to manage everyday pressures common in the healthcare sector and because of the quality of the methodology [40]. Our study using the French language version with minor adaptation to the healthcare setting showed excellent internal consistency, with Cronbach’s alpha = 0.96 for the full scale and ranging from 0.93 to 0.83 for the subscales. These were similar to values achieved with the original instrument. Data were normally distributed, and reliability levels satisfied criteria for empirical studies and were similar to those found in other studies using TR@W [40,42]. The conceptual suitability issue also emerged with the Work-SoC-9 questionnaire. The choice built upon Antonovsky’s theoretical basis of salutogenesis and its complementarity with the huge number of studies focusing on pathogenic aspects of the COVID-19 pandemic. Moreover, the influence of a volatile working environment and individual characteristics on the Work-SoC was already confirmed by Vogt et al. (2013) [31] with cross-sectional data. Our first utilization of these questionnaires in oncology teams raised endogeneity as a potential bias to our study findings [65] due to an omitted variable related to the wicked nature of adversity of work in oncology, to simultaneity related to how both main variables affect each other, to measures that may be not sufficiently sensitive to change, or to the convenience sample that meant team members on sick leave were not included in the study [66].

## 5. Conclusions

Our results suggest that the French version of these questionnaires can be used in the healthcare sector, which paves the way for future research using these tools. This is a considerable effort in the current post-pandemic climate that places multiple time and workload pressures on healthcare teams. The participants were all involved and knowledgeable of how practice changed during the COVID-19, contributing to generating real-world data, raising confidence in the results and increasing the usefulness of findings to inform decision-making. Future research could use longitudinal designs to follow the same teams over longer time periods. Such designs would increase the robustness of the relationship with context, although this might also be blurred by endogeneity.

## Figures and Tables

**Table 1:** TR@W and Work-SoC, subscale description, number of items, and Cronbach’s alpha.

			Cronbach’s α	
Component and Subscales	Subscale Description	No. of Items	Theoretical	Our Study ^1^
Resilience at Work Team Scale (TR@W)		42	0.95	0.96
Resourcefulness	Optimizing resources and processes. Focusing on continuous improvement. Building team processes that are effective and priority-oriented.	10	0.93	0.92
Robustness	Sharing common purpose, having solid intention with agility proactive about problem solving.	8	0.85	0.92
Self-care	Promoting stress management practices and identifying signs of overload. Focusing on developing a culture of self-care within the team.	7	0.87	0.93
Alignment	Sharing motivation for success. Being optimistic. Acknowledging progress and success.	4	0.88	0.92
Capability	Delivering in a changing landscape.	7	0.89	0.93
Connectedness	Cooperating, supporting each other and encouraging a sense of belonging to the team. Having a sense of belonging.	2	0.81	0.83
Perseverance	Persisting despite obstacles.	3	0.83	0.87
Work-related Sense of Coherence Scale (Work-SoC-9)		9	0.83	0.66
Comprehensibility	Perceived degree of structure, coherence and clarity of the work situation.	4	0.72	0.61
Manageability	Perceived availability of adequate resources to meet work demands.	2	0.73	0.64
Meaningfulness	Perceived degree to which the situation at work warrants commitment and involvement.	3	0.84	0.86
Perceived impact of COVID-19 (COVID-19)				
Perceived impact of adversity related to COVID-19	Visual analog scale of perceived impact of adversity related to COVID-19.	1	-	-
Quality of life at work (QoLW)				
Quality of life at work	Visual analog scale of perceived quality of life at work.	1	-	-
Labor Force Gender Index (LFGI)				
Gender index based on gender roles and institutionalized gender	Responsibility for caring for children, occupational segregation, hours of work relative to partner/spouse, and education relative to partner or spouse. To calculate the final LFGI score, values from each of the 4 categories are summed (0–10).	4	-	-

^1^ Cronbach’s alpha in the present study showing reliability of the measurement instrument in oncology.

**Table 2:** Participant characteristics (total *n* = 189).

Characteristics	Mean (SD)	Frequency ^1^	%
Age (years)	42.63 (10.28)		
Gender			
Female		152	80.42
Male		33	15.87
Prefers not to answer		4	3.17
Labor Force Gender Index (score 0–10)	4.67 (1.72)		
Education level completed			
Secondary school ^2^		10	7.87
College or CEGEP		39	30.71
University		78	61.41
Experience in the oncology team			
Less than 10 years		29	22.66
10 years or more		99	77.34
Profession			
Oncologist		21	12.88
Nurse		64	39.26
Other professional team member ^3^		56	34.36
Clerical staff		15	9.20
Other		7	4.29
Role in oncology			
Clinician (direct care only)		66	43.42
Manager (front line and direction)		11	7.24
Dual role (clinician + manager)		53	34.87
Administrative support		15	9.87
Other		7	4.61
Average hours worked per week	38.53 (8.60)		
Current living situation			
Lives with partner or spouse		92	80
Does not live with partner or spouse		16	13.91
Prefers not to answer		7	6.09

^1^ Only valid responses. ^2^ Only possible for clerical staff. ^3^ Other professional team members: pharmacists, social workers, psychologists, physical therapists, and nutritionists.

**Table 3:** Descriptive statistics and correlations between TR@W, Work-SoC-9, and control variables.

Variables (Range)	M	SD	1	2	3	4	5	6	7	8	9	10	11	12	13	14	15	16
1—TR@W (1–7)	4.87	1.09	1.00															
2—Resourcefulness	4.87	1.10	0.94 ***	1.00														
3—Robustness	4.95	1.17	0.93 ***	0.86 ***	1.00													
4—Self-care	4.35	1.44	0.89 ***	0.80 ***	0.76 ***	1.00												
5—Alignment	4.87	1.28	0.91 ***	0.79 ***	0.79 ***	0.79 ***	1.00											
6—Capability	5.42	1.18	0.91 ***	0.83 ***	0.78 ***	0.75 ***	0.86 ***	1.00										
7—Connectedness	5.19	1.38	0.85 ***	0.76 ***	0.78 ***	0.73 ***	0.83 ***	0.80 ***	1.00									
8—Perseverance	5.27	1.11	0.84 ***	0.80 ***	0.78 ***	0.64 ***	0.76 ***	0.79 ***	0.69 ***	1.00								
9—Work-SoC-9 (1–7)	5.42	1.25	0.55 ***	0.52 ***	0.52 ***	0.54 ***	0.48 ***	0.50 ***	0.46 ***	0.42 ***	1.00							
10—Comprehensibility	4.88	1.09	0.52 ***	0.51 ***	0.48 ***	0.52 ***	0.42 ***	0.45 ***	0.43 ***	0.34 ***	0.86 ***	1.00						
11—Manageability	4.64	1.06	0.31 ***	0.30 ***	0.28 ***	0.34 ***	0.28 ***	0.25 ***	0.24 ***	0.24 ***	0.68 ***	0.55 ***	1.00					
12—Meaningfulness	5.98	1.14	0.43 ***	0.37 ***	0.40 ***	0.38 ***	0.40 ***	0.44 ***	0.39 ***	0.38 ***	0.74 ***	0.39 ***	0.23 ***	1.00				
13—QoLW (0–100)	65.14	22.82	0.62 ***	0.57 ***	0.58 ***	0.64 ***	0.59 ***	0.51 ***	0.56 ***	0.46 ***	0.59 ***	0.62 ***	0.39 ***	0.37 ***	1.00			
14—Age (continuous)	42.39	9.68	−0.19 **	−0.18 **	−0.19 **	−0.13 *	−0.19 **	−0.23 **	−0.17 **	−0.11	0.03	0.01	0.09	0.01	−0.02	1.00		
15—COVID-19 (0–100)	50.81	27.55	0.03	0.00	0.00	0.01	0.07	0.07	0.05	0.02	−0.16 *	−0.17 **	−0.07	−0.07	0.00	−0.18 **	1.00	
16—LFGI (1–10)	4.08	1.31	0.14 *	0.15 *	0.12	0.11	0.13 *	0.17 **	0.11	0.11	0.16 *	0.18 **	0.14 *	0.10	0.08	−0.06	−0.09	1.00

*p*-value: * < 0.05, ** < 0.01, and *** < 0.0001. M, mean; SD, standard deviation. Note: Higher score reflects a more positive perception for all variables. Reverse scoring for negatively phrased items was used. LFGI: To calculate the final LFGI scores, the values from each of the 4 categories are summed. LFGI scores ranged from 0 to 10, with higher scores indicating more feminine gender roles. QoLW: visual analog scale, from 0 (worst score requiring intervention) to 100 (highest score). COVID-19: visual analog scale, from 0 (“no impact”) to 100 (“worst imaginable impact”).

## Data Availability

The data presented in this study are available from the corresponding author upon reasonable request.
